# Insight Into the Role of Ferroptosis in Non-neoplastic Neurological Diseases

**DOI:** 10.3389/fncel.2020.00231

**Published:** 2020-08-06

**Authors:** Jianwei Lei, Zhihua Chen, Shuxin Song, Chunpeng Sheng, Sihui Song, Jianming Zhu

**Affiliations:** Department of Neurosurgery, The Second Affiliated Hospital of Nanchang University, Nanchang, China

**Keywords:** ferroptosis, stroke, neurodegenerative disease, Alzheimer’s disease, Parkinson’s disease, iron

## Abstract

Ferroptosis is an iron-dependent form of cell death characterized by the accumulation of intracellular lipid reactive oxygen species (ROS). Ferroptosis is significantly different from other types of cell death including apoptosis, autophagy, and necrosis, both in morphology and biochemical characteristics. The mechanisms that are associated with ferroptosis include iron metabolism, lipid oxidation, and other pathophysiological changes. Ferroptosis inducers or inhibitors can influence its occurrence through different pathways. Ferroptosis was initially discovered in tumors, though recent studies have confirmed that it is also closely related to a variety of neurological diseases including neurodegenerative disease [Alzheimer’s disease (AD), Parkinson’s disease (PD), etc.] and stroke. This article reviews the definition and characteristics of ferroptosis, the potential mechanisms associated with its development, inducers/inhibitors, and its role in non-neoplastic neurological diseases. We hope to provide a theoretical basis and novel treatment strategies for the treatment of central nervous system diseases by targeting ferroptosis.

## Introduction

Ferroptosis, an iron-dependent type of programmed cell death, is different from apoptosis, autophagy, and necrosis with regards to both morphology and biochemistry (Stockwell et al., 2017). Previous studies have shown that ferroptosis is not only related to tumorigenesis (Liang et al., 2019) but also involved in the processes of various neurological diseases (Alim et al., 2019; Derry et al., 2020). However, its specific role and mechanism in tumorigenesis and neurological diseases are still unclear. This article reviews the mechanism of ferroptosis and its role in non-neoplastic neurological diseases to provide novel ideas for the development of therapeutic drugs that target ferroptosis.

## Definition and Characteristics of Ferroptosis

Dolma et al. (2003) found that the compound erastin can selectively kill RAS-mutated tumor cells, without any subsequent changes in nuclear morphology, DNA fragmentation, and other processes. Interestingly, erastin cannot be repressed by caspase inhibitors. Later, other scholars found that this ferroptosis can be inhibited by iron-chelating agents, and is accompanied by an increase in lipid reactive oxygen species (ROS) in the cell cytoplasm (Yang and Stockwell, 2008). Therefore, Dixon et al. (2012) named this form of cell death ferroptosis and defined it as an iron-dependent, non-apoptotic form of cell death characterized by intracellular lipid ROS accumulation, after which the concept was widely accepted by other researchers (Cao and Dixon, 2016; Xie et al., 2016).

Apoptosis, which refers to a process of programmed cell death dependent on caspase-3, mainly manifests as cell shrinkage, the disappearance of the mitochondrial membrane potential, chromatin concentration, and plasma membrane invagination to form apoptotic bodies (Williams and Smith, 1993). Autophagy is a process in which cells use lysosomes to degrade damaged organelles or macromolecules, The autophagy process is accompanied by the expansion of the Golgi apparatus and endoplasmic reticulum, nuclear condensation, and formation of the autophagosome (Mizushima and Komatsu, 2011). Necrosis refers to cell death that is caused by physical, chemical, or biological factors. It is characterized by swelling of the organelles, rupturing of the plasma membrane, and the release of cellular contents, all of which lead to the development of inflammation (Wallach et al., 2016). On the other hand, ferroptosis is characterized by a complete plasma membrane, increased mitochondrial membrane density, a reduced or disappeared crest, increased release of oxidized polyunsaturated fatty acids (PUFAs), and lipid ROS in the cytoplasm, all of which can be inhibited by iron-chelating agents (Stockwell et al., 2017).

## Mechanism of Ferroptosis

Iron metabolism dysfunction, intracellular lipid ROS production, and degradation imbalance contribute to the occurrence of ferroptosis.

### Iron Metabolism and Ferroptosis

Free Fe3+ in the blood forms a complex with extracellular transferrin (Tf), which binds to transferrin receptor 1 (TfR1) on the cell membrane, and forms endosomes that are transported into the cell under endocytosis. In the cell, Fe3+ is catalyzed into Fe2+ by the enzyme’s six-transmembrane epithelial antigen of the prostate (STEAP3), which is transported from the endosome to the cytosol through the divalent metal ion transporter 1 (DMT1; Koskenkorva-Frank et al., 2013). Fe2+ can be pumped out through ferroportin, which is located on the cellular membrane or stored in ferritin in the cytoplasm to achieve intracellular iron homeostasis. When excess Fe2+ is produced in the cells, lipid ROS are generated by the Fenton chemical reaction, leading to the continuous accumulation of lipid ROS within the cell and the eventual development of ferroptosis (Cao and Dixon, 2016; Xie et al., 2016).

### Lipid ROS Metabolism and Ferroptosis

The accumulation of lipid ROS is an important cause of ferroptosis, though the exact way it is produced remains to be elucidated (Muhoberac and Vidal, 2019; Stockwell and Jiang, 2020). ROS can interact with PUFAs on the lipid membrane to form lipid ROS. When a large amount of lipid ROS accumulates in the cell, it causes ferroptosis. Lipoxygenase is likely involved in the formation of iron-dependent lipid ROS, as it catalyzes the oxidation of PUFAs to lipid hydroperoxides. When numerous iron ions are present in the cytoplasm, lipid hydroperoxides form toxic lipid free radicals, causing cellular damage. In parallel, these free radicals can transfer protons near PUFAs, which starts a new round of lipid oxidation reaction and causes more serious oxidative damage (Nakamura et al., 2019; Su et al., 2019; Wang et al., 2020).

### GPX4 Regulation and Ferroptosis

GPX4 is a class of antioxidant enzymes. GSH can degrade H_2_O_2_ and lipid ROS into H_2_O, and their corresponding alcohols, respectively, thus reducing intracellular lipid hydroperoxide, and organic hydroperoxide. These reactions cause cells to avoid oxidative damage, which is essential for cell survival (Imai et al., 2017; Ingold et al., 2018; Li et al., 2020).

#### GSH Synthesis Regulation Pathway and Ferroptosis

As GSH is a necessary co-factor for the function of GPX4, its synthesis directly affects GPX4 activity. The cystine/glutamate reverse transporter on the cell membrane, system x_c_^−^, transports glutamate to the outside of the cell and cystine into the cell. Once in the cytoplasm, cystine is transformed into cysteine (Cys), which is involved in the synthesis of reduced GSH (Massie et al., 2015). When system x_c_^−^ transport cystine is blocked, the intracellular Cys levels are reduced, resulting in a decrease in GSH synthesis, a loss of GPX4 activity, intracellular lipid ROS accumulation, and ferroptosis. Inducers such as erastin can reduce intracellular Cys levels, inhibit GSH synthesis, and attenuate the accumulation of intracellular lipid ROS, leading to the development of ferroptosis by inhibiting the activity of system x_c_^−^ (Dai et al., 2020; Wang et al., 2020).

#### Mevalonate Regulation Pathway

GPX4 is a selenium-containing protease, the activity of which is affected by the catalytic center selenocysteine (Sec). The genetic code of Sec is UGA, which is the same as the stop codon. Therefore, special transport RNA (tRNA^[Ser]Sec^) is required for this process (Wirth et al., 2014; Schweizer and Fradejas-Villar, 2016). The maturation process of tRNA^[Ser]Sec^, a single tRNA that transports Sec, requires prenylation modification at a specific adenine site. This is required for Sec to participate in the synthesis of selenoproteins. The prenyltransferase uses isopentenyl pyrophosphate (IPP) as the donor, which is an important product of the mevalonate (MVA) pathway. Therefore, the activity of GPX4 is regulated by MVA (Seibt et al., 2019). Interestingly, statins can hinder the maturation of tRNA^[Ser]Sec^, which affects the synthesis of GPX4, and causes ferroptosis (Yu et al., 2017).

### Other Pathways

Other proteins regulate ferroptosis, such as the apoptosis molecule p53, which inhibits the activity of the transporter, reduces the intake of cystine and synthesis of GSH, and hinders the activity of GPX4 by inhibiting the expression of the system x_c_^−^ subunit SLC7A11. p53 also reduces the capacity of the antioxidant effect and increases lipid ROS levels, leading to ferroptosis (Kang et al., 2019; Leu et al., 2019; Ye et al., 2019; Li et al., 2020a). Also, activation of the mitogen-activated protein kinase (MAPK) pathway can induce ferroptosis in cancer cells (Gao et al., 2018; Li et al., 2018; Su et al., 2019). Thus, in cancer cells with RAS mutations, blocking RAS/RAF/MEK signal can inhibit ferroptosis caused by erastin (Imai et al., 2017). However, the specific mechanism is still not clear.

## Ferroptosis Inducer

### Systeme x_c_^−^

Systeme x_c_^−^ is a heterodimer composed of the transmembrane transporter SLC7A11 and regulatory protein SLC3A2L connected by disulfide bonds. This complex mediates cystine entry into cells and glutamate exit from the cells (Bridges et al., 2012; Massie et al., 2015). When the extracellular glutamate is present in excess, it inhibits the transfer of extra cysteine by systeme x_c_^−^, reduces intracellular Cys levels, and blocks the synthesis of GSH, resulting in the accumulation of intracellular lipid ROS and development of cell death (Tobaben et al., 2011; Kang et al., 2014; Yang and Stockwell, 2016). Previous studies have shown that after adding the ferroptosis inducer erastin, the level of intracellular radiolabeled Cys becomes significantly reduced, and the synthesis of GSH is blocked, subsequently causing ferroptosis (Dixon et al., 2012; Du et al., 2020). Sorafenib, a targeted therapeutic drug approved by the United States Food and Drug Administration (US FDA) for metastatic kidney cancer, can inhibit the activity of CRAF, BRAF, VEGFR, and PDGFR-β kinase in tumor cells (Hiles and Kolesar, 2008; Rini et al., 2020). Studies have shown that sorafenib can induce the occurrence of ferroptosis in tumor cells, opening up a novel method for tumor treatment (Sun et al., 2016; Xu et al., 2020). Sulfasalazine (SAS) is a sulfonamide antifungal drug that is widely used in the treatment of colitis. It can inhibit the expression of systeme x_c_^−^ subunit SLC7A11 and reduce the levels of intracellular Cys, leading to the induction of ferroptosis in the pancreas, breast, head, or neck cancer (Kim et al., 2018; Yamaguchi et al., 2018; Yu et al., 2019; Shin et al., 2020). Glutamate can reduce intracellular Cys expression by inhibiting the transfer of systeme x_c_^−^, which leads to the inhibition of GSH synthesis and causes ferroptosis (Song et al., 2018; Wang et al., 2019).

### GPX4 Inhibitor

While erastin acts on systeme x_c_^−^, it indirectly affects the synthesis of intracellular GSH, which, in turn, affects the activity of GPX4, causing the accumulation of intracellular lipid ROS and triggering ferroptosis (Yang et al., 2014; Hao et al., 2017). RAS selective lethal small molecule 3 (RSL3) cannot affect the synthesis of GSH in the cell, but can directly inhibit the target protein GPX4, resulting in loss of GPX4 activity and triggering ferroptosis (Sui et al., 2018; Ye et al., 2019; Vučković et al., 2020). The small molecule FIN56 promotes the degradation of the GPX4 protein and reduces the synthesis of the lipophilic antioxidant coenzyme Q 10 (CoQ 10) through the MVA pathway. This weakens the inhibitory effect of CoQ 10 on the production of lipid ROS, leading to the occurrence of ferroptosis. When GPX4 was overexpressed in cells, FIN56-induced cell death was inhibited (Shimada et al., 2016; Gaschler et al., 2018).

### GSH Depleting Agent

As a reducing agent of GPX4, GSH directly affects the activity of GPX4 and induces ferroptosis when its synthesis is blocked. Previous studies have found that, in RAS mutant cells, buthionine sulfoximine (BSO) can inhibit the GPX4 synthesis, which reduces the synthesis of GSH, inhibits GPX4 activity, leads to the accumulation of intracellular lipid ROS, and causes ferroptosis (Nishizawa et al., 2018; Zhang et al., 2018).

## Ferroptosis Inhibitor

### Iron Chelating Agent

Iron chelating agents can turn free iron ions into stable compounds, thereby reducing its toxic effects and inhibiting the process of ferroptosis. Deferoxamine (DFO), a bacterial metabolite, is one type of iron-chelating agent that can bind to the six coordinated bonds in the center of the iron atom, reduce the Fe2+ level in the cytoplasm, and inhibit lipid ROS formation caused by Fe2+ accumulation. Ultimately, DFO inhibits the occurrence of ferroptosis (Louandre et al., 2013; Ma et al., 2016; Chen et al., 2017). Another chelating agent, ciclopirox (CPX), is a classical antifungal drug. The addition of CPX to neurons *in vitro* can inhibit glutamate-induced cell death (Ma et al., 2013).

### Lipid ROS Inhibitor

Ferrostatin-1 is an aralkylamine-containing antioxidant that can lower levels of lipid ROS in the cytoplasm and inhibit ferroptosis induced by either erastin or RSL3 (Wenzel et al., 2017; Li et al., 2019). Similarly, lipoxstatin-1, which contains amide and sulfonamide subunits, can inhibit the lipid peroxidation pathway and the occurrence of ferroptosis by lowering lipid ROS levels in the cytoplasm (Skouta et al., 2014). Zileuton is a novel and selective 5-lipoxygenase inhibitor that can prevent the production of ROS in the cytoplasm and effectively prevent glutamate-induced ferroptosis in mouse hippocampal neuronal cells (Liu et al., 2015; Yuan et al., 2016). As a well-known lipid ROS inhibitor, vitamin E can reduce cell death by inhibiting the lipid oxidation pathway, thereby attenuating neurological dysfunction (Carlson et al., 2016; Imai et al., 2017; Hinman et al., 2018).

## Ferroptosis and Nervous System Diseases

Statistics show that the number of patients with neurological diseases such as stroke and neurodegenerative disease increases year by year, thus placing a great burden on families and society. However, due to the unclear pathogenesis of these diseases and a lack of reliable diagnostic and treatment methods, a greater in-depth research is urgently needed. Previous studies on ferroptosis have focused on the field of cancer. In recent years, researchers have found that ferroptosis can also exert a significant effect on neurological diseases (Yan and Zhang, 2019; Yan et al., 2020).

### Traumatic Brain Injury and Ferroptosis

Traumatic brain injury (TBI) is a leading cause of disability and mortality, with 1.5 million hospital admissions and 57,000 deaths in Europe each year. Acute brain injuries resulting from TBI can lead to lasting neurologic and cognitive problems. Mechanisms accounting for brain damage after TBI include biophysical forces, neuropathological changes (e.g., multifocal axonal injury, microglial activation, and microhemorrhages) and pathological responses (e.g., inflammation, mitochondrial dysfunction, and oxidative stress). Therapeutic strategies for TBI include pharmacological agents (e.g., anti-inflammatory agents and cell cycle inhibitors), noninvasive approaches (e.g., exercise therapy and transcranial magnetic stimulation), and biologics. Recent studies have shown that ferroptosis may play a role in TBI. After establishing a controlled cortical impact injury (CCI) mouse model, Xie et al. (2016) observed iron accumulation, reduced GPx activity, and increased lipid ROS after TBI. They also found that the administration of Ferrostatin-1 by cerebral ventricular injection reduced ferroptosis and attenuated injury lesions. The role of ferroptosis in TBI and the neuroprotective function of baicalein were supported by Li et al. (2020a) who conducted *in vivo* and *in vitro* studies and also showed that baicalein had neuroprotective effects against posttraumatic epileptic seizures. Xie et al. (2016) detected the expression of ferroptosis-related molecules at 6 h, 12 h, 24 h, 48 h, and 72 h following CCI in mice. They reported that overexpression of miR-212-5p reduced ferroptosis while downregulation of miR-212-5p induced ferroptosis partially by targeting prostaglandin-endoperoxide synthase-2. Moreover, the administration of miR-212-5p significantly improved learning and spatial memory in CCI mice. Therefore, ferroptosis participates in the pathological process of TBI, and inhibiting ferroptotic death *via* different pathways may have neuroprotective effects. Additionally, due to a damaged blood-brain barrier and impaired homeostasis, TBI can contribute to secondary injury that occurs from hours to months. The delayed period suggests a potential therapeutic window intervention. However, because of limited data, the time window is under exploration and more studies on ferroptosis associated treatment for TBI are needed.

### Stroke and Ferroptosis

Stroke has high morbidity, high lethality, and a high disability rate. Stroke can be classified into either hemorrhagic or ischemic stroke. Ischemic stroke develops due to carotid and vertebral artery stenosis or occlusion, causing the insufficient blood supply to the brain. This results in a depletion of oxygen and nutrients within the brain, leading to oxidative stress, mitochondrial damage, and ultimately, cell death (Andrabi et al., 2020; Datta et al., 2020). Previous studies have found that ferroptosis is closely related to ischemic stroke (Ahmad et al., 2014) found that in a mouse ischemic stroke model, GSH was significantly reduced, and the levels of lipid ROS were increased. Other scholars report that ferroptosis can cause neuronal death after ischemic stroke and that an inhibitor of ferrostatin-1 or liproxatin-1 plays a protective role in the mouse ischemic stroke model (Guan et al., 2019; Lan et al., 2020). Li et al. (2020a) analyzed the ultrastructure of tissue samples from patients with cerebral hemorrhage using transmission electron microscopy and observed that ferroptosis coexists with apoptosis, necrosis, and autophagy (Nikoletopoulou et al., 2013; Li et al., 2018). The combined use of inhibitors that target multiple avenues of cell death is better than the use of a certain form of death inhibitor in reducing neuronal damage (Weiland et al., 2019). Previous studies have found that selenium ions can enhance the expression of GPX4 and inhibit ferroptosis by activating the transcription factors TFAP2c and Sp1 (Ingold et al., 2018; Alim et al., 2019; Conrad and Proneth, 2020). Treatment of selenium ions can enhance the expression of the antioxidant GPX4, protect from neuronal injury, and improve behavioral defects in mouse models of stroke (Alim et al., 2019). Li et al. (2020b) found that inhibition of ferroptosis alleviates early brain injury after subarachnoid hemorrhage *in vitro* and *in vivo*, which is associated with a reduction of lipid peroxidation. Therefore, suppressing ferroptosis can be used as an effective treatment for stroke.

### Neurodegenerative Disease and Ferroptosis

#### Alzheimer’s Disease and Ferroptosis

Alzheimer’s disease (AD) is a familial neurodegenerative disease. The clinical manifestations include cognitive impairment, memory decline, executive ability decline, and personality changes, among others. The pathological features of AD include the presence of senile plaques that are formed by extracellular β-amyloid deposition, nerve fiber tangles formed by abnormal phosphorylation of Tau protein, neuronal damage, and abnormal synaptic function (Ballard et al., 2011; Palop and Mucke, 2016). Characteristics associated with ferroptosis can be detected in the brains of AD patients and mice, such as iron metabolism disorder, glutamate excitotoxicity, and lipid ROS accumulation. Numerous studies show that ferritin in the brain of AD patients is related to the content of apolipoprotein in the cerebrospinal fluid. Increased ferritin is accompanied by an up-regulation in the expression of the AD risk gene *APOE-ε4*, indicating that the iron levels in the brain affect the AD process and that high levels of iron in the brain can be used as a risk factor of AD (Ayton et al., 2015; Xu et al., 2016). Additionally, glutamate excitotoxicity is involved in the pathogenesis of AD. Dysfunction of systeme x_c_^−^ during ferroptosis can lead to an increase in the concentration of extracellular glutamate, causing excitotoxicity (Kang et al., 2018; Zille et al., 2019; Zhang et al., 2020). Furthermore, oxidative stress is critically related to AD. Recent studies have shown that oxidative stress can promote oligomeric Aβ disorder and tau protein tangles-induced neurotoxicity (Jiang et al., 2016; Luengo et al., 2019). Accumulation of lipid ROS during ferroptosis can cause oxidative damage to cells, resulting in neuronal damage and the development of AD (Xie et al., 2016; Wu et al., 2018). Hambright et al. (2017) found that a knockout of GPX4 in the mouse brain neurons caused neuronal degeneration, accompanied by cognitive dysfunction. However, administration of the ferroptosis inhibitor, Liproxstatin-1, can reverse the neuronal degeneration and improve the cognitive function of mice (Hambright et al., 2017).

#### Parkinson’s Disease and Ferroptosis

Parkinson’s disease (PD) is a degenerative neurological disease that is mainly observed in the elderly population. The clinical manifestations include resting tremor, bradykinesia, muscle stiffness, and posture and gait disorders, accompanied by memory loss, mental decline, and emotional disorders. The pathological features of the disease mainly include degeneration and death of midbrain substantia nigra dopaminergic (DA) neurons, reduced striatum DA concentration, and formation of Lewy bodies (Goedert and Compston, 2018; Homayoun, 2018). Recently, researchers found that in PD patients, iron and hydroxyl radical levels in the substantia nigra are increased, leading to damage of DA neurons. Besides, other ferroptosis characteristics such as GSH depletion have also been observed in the substantia nigra of PD patients (Zhao, 2019; Devos et al., 2020; Zhang et al., 2020). Another study reported that loss of plasma ceruloplasmin iron oxidase activity in the substantia nigra of PD patients, which led to the accumulation of iron peroxide. Interestingly, iron chelating agents were able to reverse the accumulation of iron ions caused by the absence of ceruloplasmin, improve the exercise ability of PD mice, and effectively reduce any neurological damage caused by MPTP (Ayton et al., 2013), consistent with prior results performed in PD patients (Grolez et al., 2015). Do Van et al. (2016) and Dächert et al. (2020) confirmed using *in vitro* brain slice examination and *in vivo* MPTP mouse model that PKCα activation caused MEK activation, which, in turn, caused ferroptosis. Therefore, iron chelators, ferrostatin-1, and PKC inhibitors, which regulate ferroptosis, may represent novel drugs for PD patients.

#### Huntington’s Disease and Ferroptosis

Huntington’s disease (HD) is a type of inherited neurological disease caused by repeated amplification of CAG in the *HTT* gene. The clinical manifestations of HD are involuntary dance-like movements, dementia, and mood disorders (Roos, 2010; Wyant et al., 2017). Ferroptosis is related to the excitotoxicity of glutamate and GSH-mediated redox reactions in HD. Abnormal levels of glutamate, GSH, iron ions, and accumulation of intracellular lipid ROS have also been detected in HD patients (Dubinsky, 2017; Kumar et al., 2020). In animal HD models, characteristics of ferroptosis, including blocked GSH synthesis and decreased activity of GPXs, have been detected (Ribeiro et al., 2012; Mason et al., 2013). Treatment of HD rats using the ferroptosis inhibitor ferrostatin-1 was found to inhibit lipid peroxidation and reduce neuronal death. However, the use of iron chelating agents can reverse the changes of lipid peroxidation inhibition and neuronal death reduction, and significantly improve cognitive dysfunction of HD rats (Chen et al., 2013; Skouta et al., 2014; Agrawal et al., 2018).

### Periventricular Leukomalacia and Ferroptosis

Periventricular leukomalacia (PVL), a secondary brain white matter injury, is commonly seen in premature infants and surviving children with postpartum asphyxia. Hypoxia before and after the perinatal period causes ischemia of the peripheral ventricle region of the child, resulting in damage to neurons and oligodendrocytes (OLs) and abnormal formation of the myelin sheath. Hypoxia also causes softening of the white matter around the ventricles, resulting in bilateral spastic hemiplegia, quadriplegia, and mental retardation (Deng et al., 2008; Back, 2017). Indirect evidence has indicated that ferroptosis may play a key role in the development of PVL. Inder et al. (2002) found a large number of lipid oxidation products in the cerebrospinal fluid of infants with PVL. Furthermore, OLs death in PVL is related to lipid peroxidation, which is one of the key characteristics of ferroptosis. These results suggest that ferroptosis may be involved in the pathogenesis of PVL (Inder et al., 2002). Similarly, other studies that used cultivated OLs in cysteine-free medium found that exhaustion of GSH caused ferroptosis in cells, and the administration of ferrostatin-1 can effectively prevent ferroptosis in OLs (Novgorodov et al., 2018; Nobuta et al., 2019).

## Conclusions and Prospects

**Figure 1:**
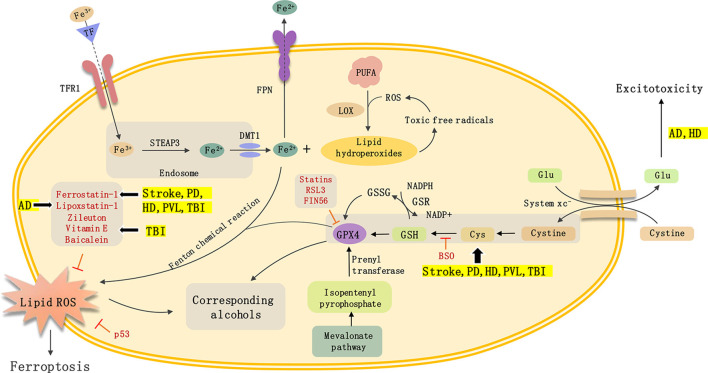
Mechanisms of ferroptosis and non-neoplastic neurological diseases.

In this review article, we mainly describe the role of ferroptosis in non-neoplastic neurological diseases, including TBI, stroke, neurodegenerative disease, and PVL (Figure 1). And summarize the inducer and inhibitor of ferroptosis used in previous research. Ferroptosis is an iron-dependent form of cell death characterized by the accumulation of intracellular lipid ROS. Ferroptosis is closely related to the progress of various neurological diseases including AD, PD, HD, and stroke. However, there are still many issues in this field that need to be resolved. First, researchers need to examine the relationship between ferroptosis and other cell death types such as apoptosis, autophagy, and necrosis. Second, the similarities and differences in the molecular mechanism of ferroptosis across different disease states need to be better defined. Third, there needs to be further research on drugs that target ferroptosis to determine whether they can play an important role in the clinical treatment of neurological diseases. In-depth research on ferroptosis will help us further clarify the pathogenesis of neurological diseases and provide a theoretical basis for clinical treatment targeting ferroptosis to prevent and treat neurological diseases.

## Author Contributions

All authors participated in analyzing and discussing the literature, commenting on, and approving the manuscript. JZ supervised the research, led the discussion, wrote and revised the manuscript. All authors contributed to the article and approved the submitted version.

## Conflict of Interest

The authors declare that the research was conducted in the absence of any commercial or financial relationships that could be construed as a potential conflict of interest.
